# A Combined Synthetic-Fibrin Scaffold Supports Growth and Cardiomyogenic Commitment of Human Placental Derived Stem Cells

**DOI:** 10.1371/journal.pone.0034284

**Published:** 2012-04-03

**Authors:** Antonella Lisi, Enrica Briganti, Mario Ledda, Paola Losi, Settimio Grimaldi, Rodolfo Marchese, Giorgio Soldani

**Affiliations:** 1 Institute of Translational Pharmacology, National Research Council (C.N.R.), Roma, Italy; 2 Institute of Clinical Physiology, National Research Council (C.N.R.), Massa, Italy; 3 Research Center, S. Peter Hospital FBF, Rome, Italy; National Cancer Institute, United States of America

## Abstract

**Aims:**

A potential therapy for myocardial infarction is to deliver isolated stem cells to the infarcted site. A key issue with this therapy is to have at one's disposal a suitable cell delivery system which, besides being able to support cell proliferation and differentiation, may also provide handling and elastic properties which do not affect cardiac contractile function. In this study an elastic scaffold, obtained combining a poly(ether)urethane-polydimethylsiloxane (PEtU-PDMS) semi-interpenetrating polymeric network (s-IPN) with fibrin, was used as a substrate for in vitro studies of human amniotic mesenchymal stromal cells (hAMSC) growth and differentiation.

**Methodology/Principal Findings:**

After hAMSC seeding on the fibrin side of the scaffold, cell metabolic activity and proliferation were evaluated by WST-1 and bromodeoxyuridine assays. Morphological changes and mRNAs expression for cardiac differentiation markers in the hAMSCs were examined using immunofluorescence and RT-PCR analysis. The beginning of cardiomyogenic commitment of hAMSCs grown on the scaffold was induced, for the first time in this cell population, by a nitric oxide (NO) treatment. Following NO treatment hAMSCs show morphological changes, an increase of the messenger cardiac differentiation markers [troponin I (TnI) and NK2 transcription factor related locus 5 (Nkx2.5)] and a modulation of the endothelial markers [vascular endothelial growth factor (VEGF) and kinase insert domain receptor (KDR)].

**Conclusions/Significance:**

The results of this study suggest that the s-IPN PEtU-PDMS/fibrin combined scaffold allows a better proliferation and metabolic activity of hAMSCs cultured up to 14 days, compared to the ones grown on plastic dishes. In addition, the combined scaffold sustains the beginning of hAMSCs differentiation process towards a cardiomyogenic lineage.

## Introduction

Myocardial infarction (MI) is the most common cause of death and disability worldwide. Extensive loss of cardiomyocytes, substituted by scarred tissue, is the key pathological mechanism leading to left ventricle (LV) dilation and dysfunction and finally to post infarction heart failure. The use of exogenous cells to replace lost cardiomyocytes is a potential therapy to prevent cardiac remodeling and to improve LV function after MI, as it has been demonstrated in animal models and in clinical trials by transplanting mesenchymal stem cells (MSCs) into the infarcted area [1]–[5].

However, the conventional cell delivery by injection into the infarcted area is often limited by a low cell engraftment [6], [7] and an inhomogeneous cell delivery, leading to a spotty distribution of cells within the myocardial scar [4].

The implantation of cellularized scaffolds directly onto the infarcted area potentially overcomes the significant loss of cells from the site of injury following transplantation [8], [9]. Simpson and colleagues [10] demonstrated that the delivery of human MSCs by a collagen hydrogel, directly applied on the epicardial surface of the infarction, reduces myocardial remodeling. Liu et al. [11] showed that a fibrin patch seeded with MSCs surgically implanted onto necrotic areas improved LV contraction, and prevented LV dilation and heart failure. Recently, Xiong and colleagues [12] demonstrated that the transplantation of vascular cells derived from human embryonic stem cells by a fibrin 3D porous scaffold, resulted in a significant engraftment and LV functional improvement.

However, the poor mechanical properties of these biopolymer-based cell delivery systems may restrict their field of use. For cardiac regeneration it will be necessary to develop a cell delivery system which, besides being able to support cell proliferation, may also provide handling and elastic properties which do not affect cardiac contractile function. To this end we developed a combined scaffold, constituted by a fibrin layer able to sustain cell growth and differentiation, and by a microporous synthetic layer made of poly(ether)urethane-polydimethylsiloxane (PEtU-PDMS) semi-interpenetrating polymeric network (s-IPN) able to mechanically reinforce the fibrin layer providing, at the same time, suitable elastic properties to the whole scaffold.

Regarding the stem cells that can be used for cardiac regeneration, MSCs seem the most appropriate cell type to use, since due to their multilineage potential they can differentiate into a variety of cell types including cardiomyocytes and vascular endothelial cells [13], [14]. In terms of stem cell supply, the term placenta constitutes a very reliable rich source of fetal MSCs that can be kept even after a consistent number of passages (5–10). These cells, named human amniotic mesenchymal stromal cells (hAMSCs), are capable of differentiating into multiple different cell types and have immunological properties that suggest their use in an allogenic transplantation setting. In this respect, it is important to remember that placenta has a fundamental role in maintaining fetomaternal tolerance and, therefore, the immunomodulatory properties of these cells have been investigated with the aim of exploring their applicability in cell therapy-based treatments. The low immunogenic properties of hAMSCs are partially explained by their low or limited levels of HLA-ABC, and by the fact that they do not express HLA-DR and co-stimulatory molecules [15]–[18]. Their recovery do not involve any invasive procedures for the donor and their use does not create any ethical issue. In addition, the fact that placenta is generally discarded after birth and is available in large supplies, makes hAMSCs an excellent candidate for their eventual use in cell therapy approaches [15], [19], [20].

Based on these considerations we investigated the feasibility of using hAMSCs seeded on the fibrin side of the combined s-IPN PEtU-PDMS/fibrin scaffold for a potential delivery of these cells to necrotic myocardial areas. In this study we report, for the first time concerning the hAMSCs population, the results about their growth and the beginning of cardiomyogenic commitment, following nitric oxide (NO) treatment, on this scaffold.

## Results

### Light microscopy

**Figure 1:**
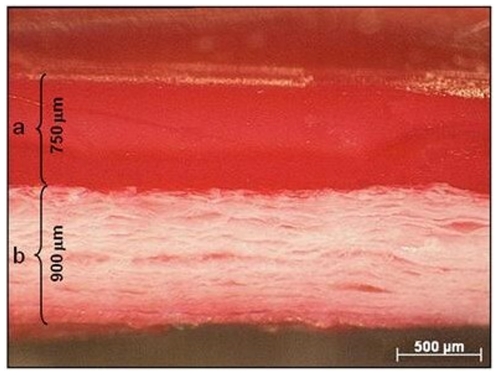
Stereo-microphotographs of the cross-section of the fibrin-based scaffold after Ponceau red staining (O.M. 30×). a) the fibrin layer shows a thickness of approximately 750 µm, b) the s-IPN PEtU-PDMS layer shows a thickness of approximately 900 µm. The two layers appear firmly attached.

The stereo-microscopical observation of the s-IPN PEtU-PDMS/fibrin combined scaffold cross-section, after Ponceau red staining, showed the presence of a homogeneous fibrin layer covering the synthetic surface. The fibrin layer exhibited a deep and even red coloration; on the contrary the s-IPN PEtU-PDMS layer does not uptake the dye and appears white. The thicknesses of the fibrin and the s-IPN PEtU-PDMS layers, evaluated by images analysis, were about 750 µm and 900 µm respectively (Fig. 1).

### Scanning electron microscopy (SEM)

**Figure 2:**
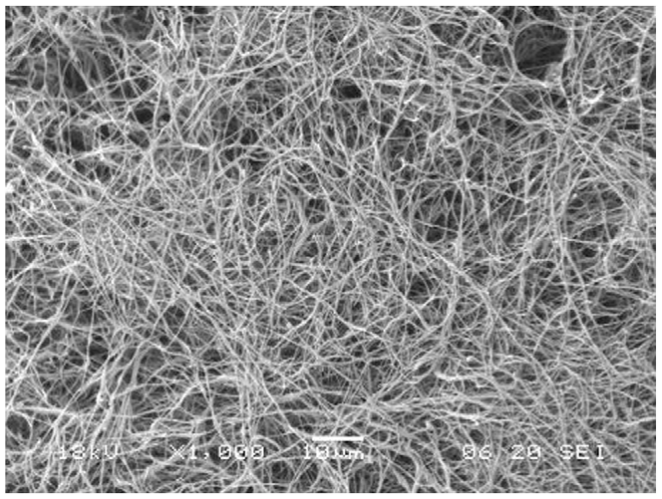
SEM-microphotographs of the fibrin side of the scaffold. The fibrin nanofibers appear randomly oriented and completely covering the underlying s-IPN PEtU-PDMS material (O.M. 1000×).

SEM microphotographs of the fibrin side of the s-IPN PEtU-PDMS/fibrin combined scaffold showed a fibrin network composed of randomly oriented nanofibers that completely covers the underlying s-IPN PEtU-PDMS material (Fig. 2). The average fibers diameters, measured by software Axiovision Rel. 4.6 (Carl Zeiss), was about 130 nm.

### Metabolic activity of hAMSC grown on fibrin combined scaffold or Petri dish and differentiated with NO

**Figure 3:**
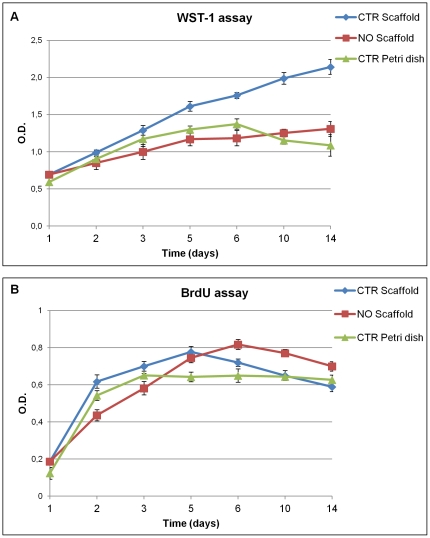
WST-1 and BrdU incorporation assays. (A) The WST-1 assay performed on hAMSCs cultured on the fibrin-based scaffold (Control Scaffold = CTR Scaffold) showed a higher metabolic activity than hAMSCs cultured on Petri dishes (Control Petri dish = CTR Petri dish). The differentiated cells cultured on the fibrin-based scaffold treated with NO (NO Scaffold) showed a decrease in metabolic activity. (B) The BrdU pulse-labelling time-course performed on hAMSCs cultured on CTR Scaffold showed a higher and more rapid proliferation rate up to 5 days, in comparison with cells cultured on CTR Petri dish. After that the cell proliferation rate on CTR scaffold slowly decrease up to reach the same value of the CTR Petri dish at 10–14 days. Cells treated with NO (NO Scaffold) decrease their proliferation rate from days 2 up to 5, while from days 6 up to 14 showed a higher proliferation rate, in comparison with the control cells.

hAMSCs treated (NO curve) or not treated (CTR curves) with SNAP, were cultured on the s-IPN PEtU-PDMS/fibrin combined scaffold and plastic Petri dishes for up to 14 days. hAMSCs grown on the scaffold surface showed a significant increase of the metabolic activity compared to the cells cultured in the Petri dish, as assessed by WST-1 assay at different time points. We also found a low metabolism in these cells treated with the differentiating agent NO (Fig. 3A) by the WST-1. These results highlight a better cell-friendly environment of this scaffold compared to Petri dishes.

### Proliferation rates of hAMSC grown on fibrin combined scaffold or Petri dish and differentiated with NO

Exponentially growing human amniotic mesenchymal stromal cells were grown on the s-IPN PEtU-PDMS/fibrin combined scaffold and plastic Petri dish up to 14 days. These cells revealed a higher metabolism corresponding to a higher proliferation rate as assessed by 5-bromo-2′-deoxy-uridine (BrdU) assay. Accordingly hAMSC cultured on the fibrin combined scaffold (CTR scaffold) showed a higher and more rapid proliferation rate up to 5 days, in comparison with the CTR Petri dish. After that the proliferation rate slowly decrease up to reach the same value of the CTR Petri dish at 10–14 days (Fig. 3B). Moreover, their differentiation by exposure to NO, as expected, showed a decrease in cell proliferation rate up to 5 days and an increase up to 14 days, in comparison with CTR scaffold. From the above results it is possible to state that the proliferation rate of hAMSCs grown on the scaffold is better compared to the one of the cells grown on Petri dishes, confirming the biocompatibility of this scaffold.

### mRNA expression of hAMSC grown on fibrin combined scaffold and differentiated with NO

**Figure 4:**
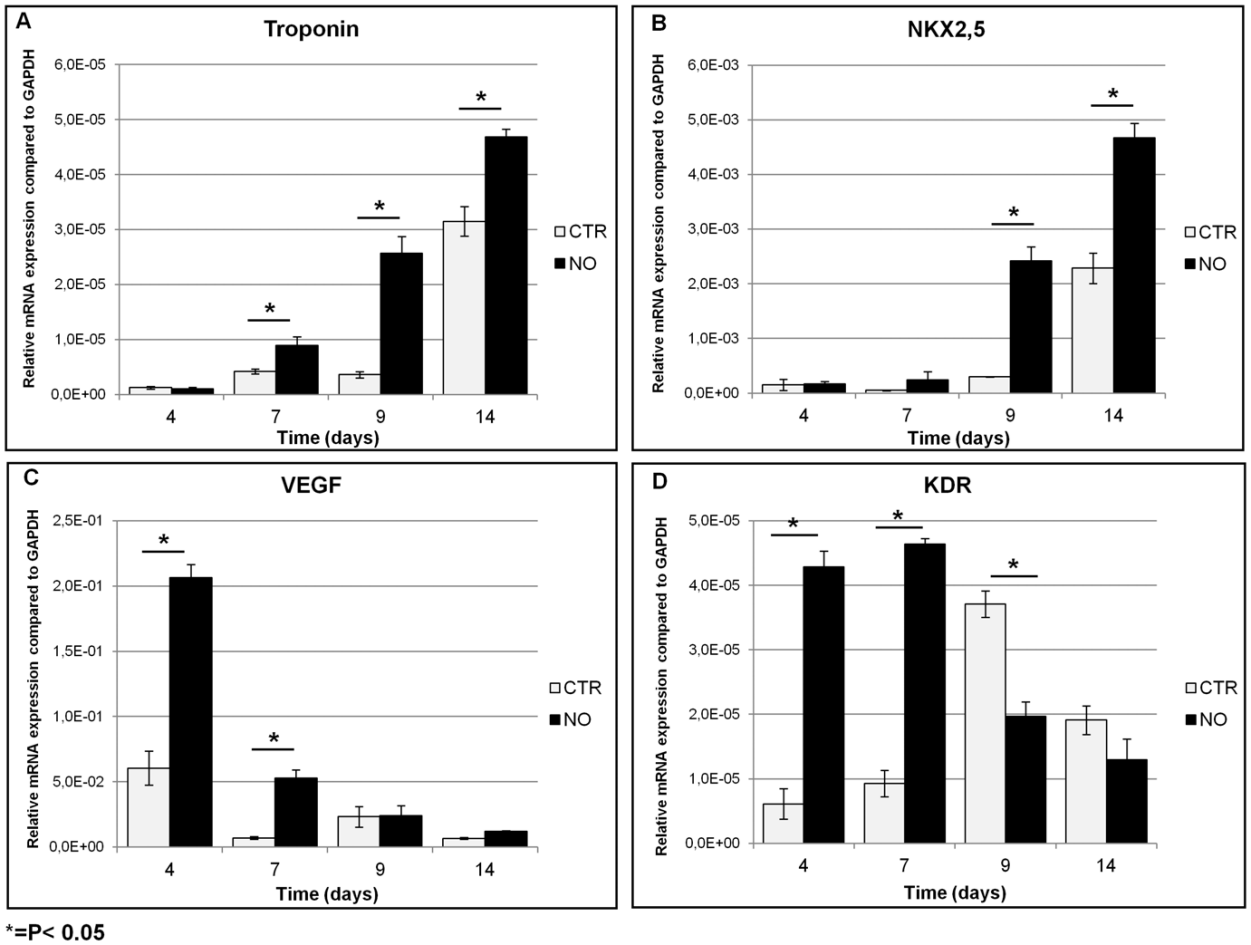
Quantitative real-time polymerase chain reaction in cardiac and vascular differentiation markers. A statistical significant increase in relative Nkx2.5, and TnI mRNA levels in NO treated cells (NO) compared to control cells (CTR) at 7, 9 and 14 days was shown (A, B). A high level of VEGF expression at 4 and 7 days followed by a time-related reduction at 9 and 14 days in KDR mRNA levels in NO treated cells (NO) compared to control cells (CTR) was also detected (C, D). *P<0.05.

The differentiation status of the NO-exposed cells was further assessed by examining mRNA levels for cardiac and vascular specific genes. NO treatment of hAMSCs grown on the s-IPN PEtU-PDMS/fibrin combined scaffold showed a significant increase in the expression of cardiac markers, and an opposite trend in the expression of vascular markers during the later part of the experiment (Fig. 4). RT-PCR analysis revealed a statistically significant increase at 7, 9 and 14 days in the relative Troponin I (TnI) expression and at 9 and 14 days for Nkx2.5 expression compared to the control cells (Fig. 4 A,B). The vascular differentiation markers such as Vascular endothelial growth factor (VEGF) and VEGF receptor (kinase insert domain receptor, KDR) showed a modulation trend with an early increase in their expression. VEGF and KDR mRNAs remained high up to 7 days and then slowly decreased at 9 and 14 days compared to control cells (Fig. 4 C,D). It is important to note that from the fourth to the seventh day of the experiment a greater level of both cardiac and vascular differentiation markers were found in the NO treated cells. The NO treatment is able to induce in hAMSCs grown on the scaffold a modulation of both cardiac and vascular mRNA markers.

### Vimentin and actin expression and distribution of hAMSC grown on fibrin combined scaffold and differentiate with NO

**Figure 5:**
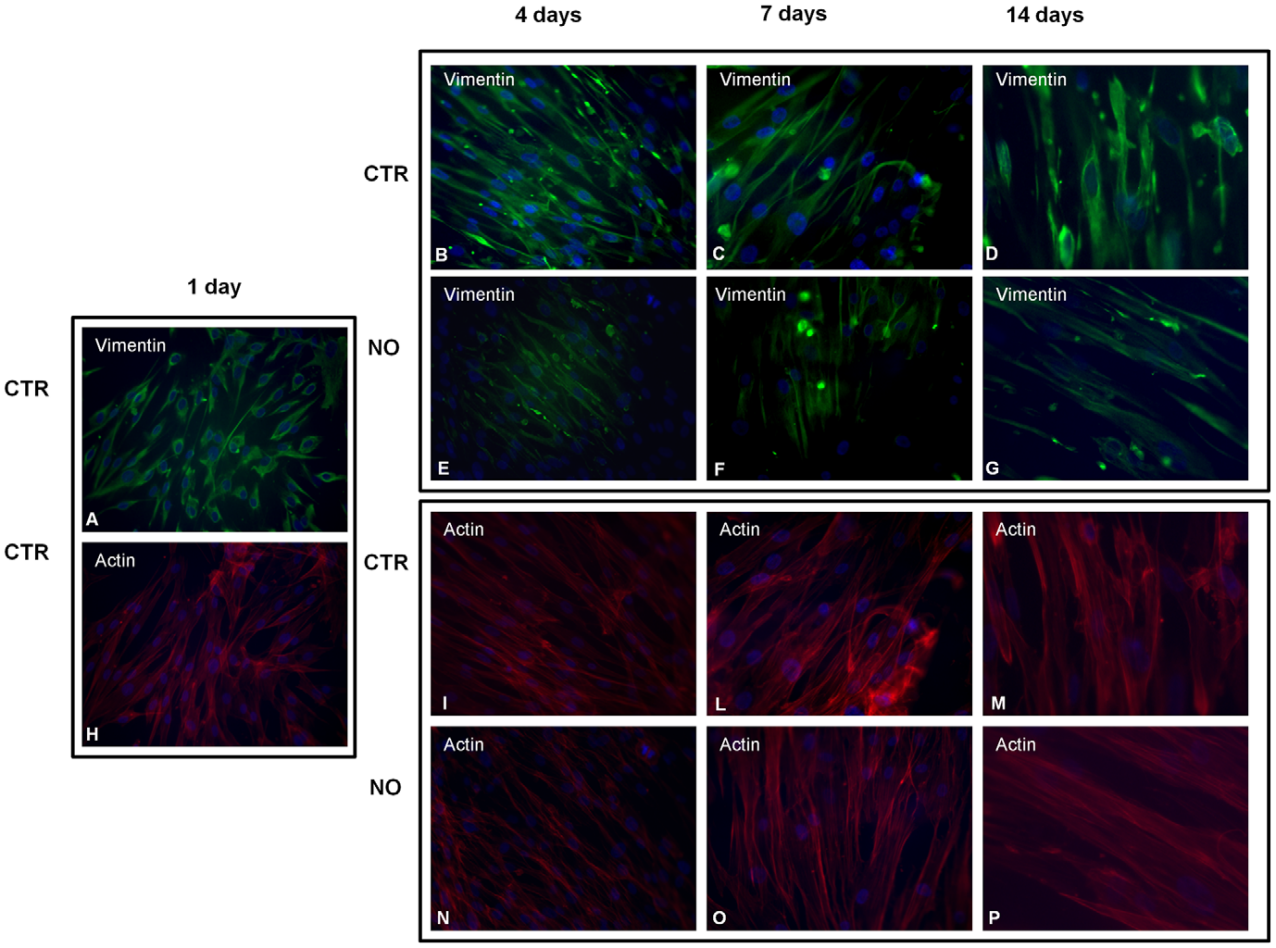
Immunofluorescence analyses of vimentin and actin filaments expression of hAMSCs cultured on the fibrin-based scaffold. Examination of vimentin immunostaining (green hue) and marking of nuclei with Hoechst (blue hue) in the hAMSCs grown on the fibrin–based scaffold whether treated (Differentiated cells; E,F,G) or not (Control cells; A,B,C,D) with NO at 1, 4, 7 and 14 days of culture (O.M. 200×). Examination of F-actin with phalloidin (red hue) and nuclei with Hoechst staining (blue hue) on the hAMSC grown on the fibrin-based scaffold (Differentiated cells; N,O,P) or not (Control cells; H,I,L,M) with NO at 1, 4, 7 and 14 days of culture (O.M. 200×).

**Figure 6:**
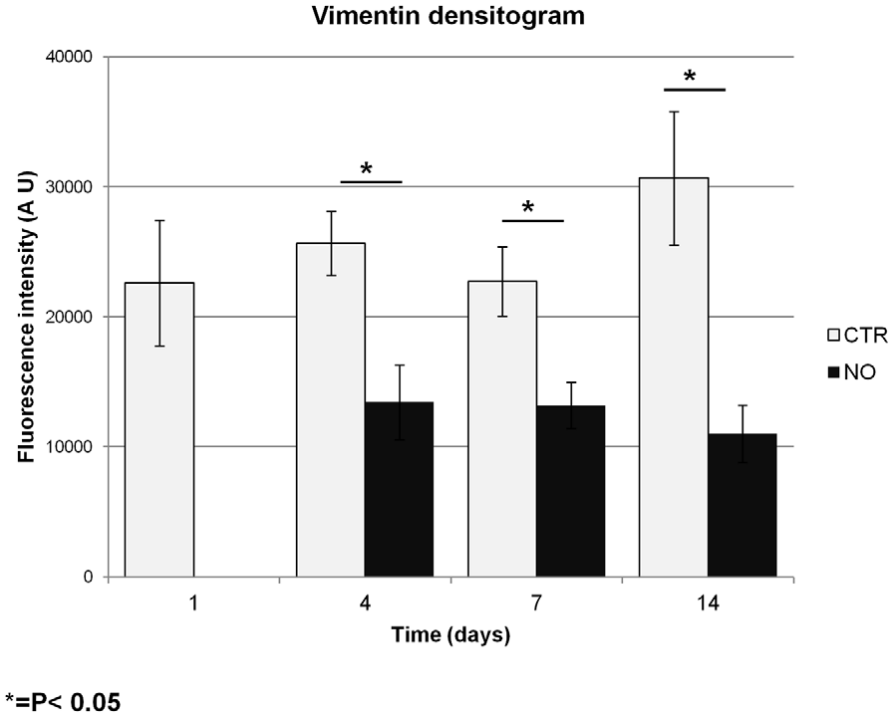
Densitogram analysis. Quantification of vimentin fluorescence intensity, by Imagej Software, showed a decrease in vimentin protein expression in the hAMSCs treated with nitric oxide (NO) compared to the control cells (CTR). Statistical evaluation of experimental data was performed with Student's *t*-test with P<0.05 as the minimum level of significance.

Vimentin (57 kDa) is an ubiquitous intermediate filament protein expressed in a wide variety of MSCs types and detected at a high level in immature stem cells and at a low level in fully differentiated stem cells. By indirect immunofluorescence analysis we observed a decrease in the vimentin protein expression in the hAMSCs growing on the s-IPN PEtU-PDMS/fibrin combined scaffold treated with NO (Fig. 5 E,F,G) compared to control cells (CTR) (Fig. 5 A,B,C,D). Densitogram analysis revealed a statistical significant reduction of vimentin fluorescence intensity at 4, 7 and 14 days of culture, while the hAMSCs were differentiating compared to the intensity recorded by the control cells (Fig. 6).

We also investigated the effect of NO treatment on cytoskeleton organization in hAMSCs growing up on the s-IPN PEtU-PDMS/fibrin combined scaffold. The fluorescence analysis of filamentous actin (F-actin) stained by Phalloidin showed that the actin filament organization on control cells was homogenously distributed on the full cellular body (Fig. 5 H,I,L,M) and maintained for all 14 days. Instead in the differentiating hAMSCs the actin network was highly concentrated around the periphery of the cell and formed unidirectional long streaked myofibrils grouped in large bundles (Fig. 5 N,O,P). The actin reorganization and the vimentin reduction suggest a beginning of cardiomyogenic commitment through NO treatment in hAMSCs.

## Discussion

Reconstituting infarcted tissue with cells capable of performing the functions of the heart or providing beneficial trophic factors for native cells is an attractive solution for myocardial repair [23]–[25]. To reconstitute the myocardium, a large number of cells need to be delivered efficiently by a cell delivery system. In this study, we tested the feasibility of a tissue-engineered approach for the potential delivery of hAMSCs to an infarcted myocardium. In particular, for the first time, hAMSCs were grown on a s-IPN PEtU-PDMS/fibrin combined scaffold and treated with NO as differentiating agent towards a cardiomyogenic lineage.

In this respect it is worth to mention that several tissue engineering approaches have already been undertaken for cardiac cell replacement therapy [26]–[34]. These include the use of biomaterial-based cellular patches to restore myocardial function. Biomaterials that have been investigated as potential scaffolds for cardiovascular tissue engineering applications can be broadly classified into categories of synthetic or naturally derived substances. Unfortunately, none of these meet the characteristics of an ideal scaffold. Common scaffold materials, ranging from biological materials such as decellularized xenogenic or allogenic matrices [35], [36], fibrin and collagen [37], [38] to synthetic polymers such as polyglycolic acid (PGA), polylactic acid (PLA), polyhydroxybutrate (PHB) as well as co-polymers of PLA acid and PGA [39], [40] meet some but not all of the requirements for an ideal scaffold material.

Among the biological materials, fibrin gel, which is a naturally occurring biodegradable matrix, has many inherent properties that make it an ideal natural biological scaffold for tissue engineering applications. Fibrin-based biomaterials are biocompatible and biodegradable and have high affinity to various biological surfaces [41]. Being a naturally occurring physiological scaffold, it supports angiogenesis and tissue repair [42].

The ability of fibrin to stimulate and support the growth of new blood vessels is well documented and plays an important role in re-establishing blood supply to ischemic tissues [43], [44]. In addition, fibrin naturally contains sites for cellular binding, and it has been shown to have excellent cell seeding effects and good tissue development [45]. However, the most significant drawback of fibrin gels is that they are structurally too weak to withstand dynamic physiological environments in vivo [46].

Therefore, the very poor mechanical properties of fibrin gel encourages researchers to combine fibrin with synthetic materials in order to obtain scaffolds with appropriate tensile strength, elasticity and handling for applications in the ischemic heart [47].

The concept of a scaffold which combines the advantages of natural and synthetic materials is very attractive and seems effective [48]–[50]. The skeleton of the synthetic polymers defines the gross shape and size of the engineered tissue and supports the forming tissue during the initial stages, whereas the fibrin gel facilitates cell invasion and growth. However, major drawbacks of biodegradable polyester used up to now (such as PGA, PLA, PHB and their co-polymers) include their inherent hydrophobicity and rigidity before implantation, lack of natural adhesive proteins and specific ligands, and the difficulty encountered in the process of creating balance between the matrix degradation time and the reconstruction of a new matrix [51].

A special concern is the stiffness of these materials which restrict their use for cardiac applications where, on the contrary, it is necessary to have synthetic polymers with high flexibility and elasticity.

Towards this end, our approach focused on the development of an elastic scaffold composed of a fibrin layer and a microporous synthetic layer made of a slowly biodegradable material, such as the PEtU-PDMS (Fig. 1). The combined scaffold was manufactured in the form of a flat membrane by a spray, phase-inversion technique in an original way, depositing simultaneously the PEtU-PDMS material and a thrombin solution over a rotating mandrel [52]. Afterwards, the thrombin incorporated in the deposited material reacts with fibrinogen to form a nanostructured fibrin layer firmly attached on the top of the synthetic layer, as previously demonstrated by peel-out test [22]. The s-IPN PEtU-PDMS/fibrin combined scaffold can be tailored with elastic modulus and mechanical strength matching approximately those of the human myocardium at the end of diastole [22]; it is biocompatible and evokes a limited inflammatory response, as already demonstrated by in vitro and in vivo experiments [21], [22], [53], [54].

Regarding stem cell to be used to reconstitute a damaged heart, recently an increasing number of scientific papers highlight that MSCs may secrete a large amount of angiogenic, antiapoptotic, and mitogenic factors (trophic factors) that are able to improve cardiac function and reduce scar formation when transplanted in animal models of MI [55]–[58]. However, in spite of the abovementioned properties, MSCs therapeutic potential for cardiac repairing is limited by age, disease states of patients and also by the low cardiac lineage commitment. An important goal of cardiac cell therapy is the identification of multipotent cells transplantable in allogenic contexts, and committable through cardiac and vascular lineages. In this context hAMSCs represent a more suitable source that could be used in cardiac regenerative medicine. Regarding the use of NO to stimulate cell cardiac commitment, it has been shown that NO is a free radical signaling molecule that regulates several differentiation processes including cardiomyogenesis [59], [60], [61]. The treatment of embryonic stem cells with NO donors such as S-nitroso-N-acetyl-D,L-penicillamine (SNAP) or 2-(N,N-diethylamino)-diazenolate-2-oxide (DEA/NO), or the transduction with the iNOS gene increases both the number of spontaneously contracting cell clusters and the expression of cardiac myosin light chain (MLC) protein, an effect blocked by NOS inhibitors [59].

The first approach for the selection of a suitable scaffold for tissue engineering should be the evaluation of its *in vitro* cell culture biocompatibility as well as its cytotoxicity [20], [21], [53], [54]. We found no evidence of cellular toxicity, but on the contrary the results of our study confirmed the biocompatibility and cell-friendly property of this scaffold. In particular hAMSCs showed a constant and steady cellular growth and a higher metabolic activity when cultured on fibrin combined scaffold (Fig. 3 A, B). Following NO treatment differentiated hAMSCs cultured on fibrin combined scaffold showed a longer lasting exponential phase, suggesting cardiomyogenic commitment and after that a restart of their proliferation (Fig. 3B). The nanostructured fibrin network (Fig. 2) on top of the scaffold provides a fine tridimensional matrix that accordingly enables a tridimensional cell growth. The fibrin network allows hAMSCs cytoskeleton reorganization (Fig. 5 N,O,P), a decrease of the vimentin mesenchymal staminality marker expression (Fig. 5) and, at 4 days of NO treatment, the beginning of cardiomyogenic commitment (Fig. 4).

We also demonstrated that hAMSCs showing a vascular lineage, when grown on a s-IPN PEtU-PDMS/fibrin combined scaffold, increase the expression of early and late cardiac differentiation markers if treated with NO agent. In hAMSCs the cardiac differentiation marker expression obtained as a long-lasting response was suggested by the persistence of markers (up to 14 days) even after NO removal. We reported a statistical significant increase in the expression of cardiac Troponin I and Nkx2.5 (Fig. 4 A, B) genes and at the same time a statistical significant decrease in the expression of vascular VEGF and KDR genes which is time-dependent on s-IPN PEtU-PDMS/fibrin combined scaffold (Fig. 4 C, D), this result is in accordance to previous study that demonstrated that physical properties of polymer scaffold can direct stem cells differentiation to cardiomyogenic lineage [62]. Interestingly, in the early phase of the NO treatment, these cells also showed a high expression of vascular markers (at day 3) that gradually decreased and highlighted, in the middle phase (days 4–7), a good level of both cardiac and vascular gene expressions suggesting their use for cardiac engrafting.

A dynamic remodeling of actin filaments and a decrease in the vimentin protein expression was also reported during NO treatment (Fig. 5 and 6). The cytoskeleton structure plays a fundamental role in the cell survival, migration, division and differentiation process for different cell types [63], [64], and also in the differentiation of mesenchymal stem cells [65], [66].

In our study hAMSCs undergo actin cytoskeletal changes during cardiomyogenic commitment. Control hAMSCs show more stress fibers with a thinner actin network distributed throughout the cytoplasm and as cardiac differentiation progresses they are replaced by some thick actin fibers principally sited in the region of the cell membrane, a typical characteristic of cardiomyocyte cells. In particular, after only 4 days of NO exposure the actin cytoskeleton rearranges and forms large bundles that run along the longer axis (the periphery of the cell) where they are more abundant and concentrated (Fig. 5 N,O,P) compared to the actin network of the undifferentiated cells (Fig. 5 H,I,L,M). Cardiac cells are subjected to mechanical strains and deformations without loss of shape or stability thanks to an elastic and compliant cytoskeleton - for this goal a thin and dense actin network is ideally adequate, as suggested by numerous theoretical models [67]–[70]. A drastic change in mechanical properties is due to the specific functions performed by cardiac cells, so the actin cytoskeletal reorganization acquired by the hAMSCs is also an important factor that suggests the achievement of a cardiomyogenic commitment.

Vimentin protein, a marker of mesenchymal staminality, is often expressed at a high level in undifferentiated stem cells where it regulates cell growth and differentiation [71]. hAMSCs treated with NO show a decreased vimentin protein expression (Fig. 5 E,F,G) compared to control cells, while the mRNAs of cardiac differentiation markers increase.

The results of this study confirm the cardiomyogenic commitment effect of the NO treatment on hAMSCs grown on the s-IPN PEtU-PDMS/fibrin combined scaffold.

In order to evaluate cardiomyocytes-derived hAMSCs capability to synchronously contract on s-IPN PEtU-PDMS/fibrin combined scaffold, we are going to develop an *in vitro* tool, similar to the one described by Katare et al. [71], able to apply a mechanical stretching to the engineered scaffold.

In conclusion we demonstrated that hAMSCs showing a vascular lineage, when grown on the fibrin side of a cell-friendly s-IPN PEtU-PDMS/fibrin combined scaffold and treated with NO, increase the expression of early and late cardiac differentiation markers, and therefore may represents a new cell delivery system that could be effectively used in cardiac regenerative medicine.

Furthermore, we can speculate that using this PEtU-PDMS/fibrin combined scaffold as a cell delivery system, the cells would be provided with a firm, but also flexible and elastic support, allowing direct application over the infarcted areas without cell loss, and providing a more site-directed repair action. Scaffold components maybe designed in a way that an engraftment may be expected in the cardiac tissue while the scaffold slowly degrades over time.

## Materials and Methods

### Fabrication of fibrin-based scaffold

The medical-grade, aromatic poly(ether)urethane (PEtU) was purchased from Lubrizol Advanced Materials, Inc. Cleveland, OH, USA (Estane® 5714) and the diacetoxy silyl terminated (tetraacetoxy functional) polydimethylsiloxane (PDMS) from United Chemical Technologies, Inc. Bristol, PA, USA. The s-IPN PEtU-PDMS containing 30% of PDMS was synthesized according to a previously described protocol [21].

Fibrinogen and thrombin were purified from human plasma by the supplier (KEDRION S.p.A., Castelvecchio Pascoli, Lucca, Italy). Lyophilized fibrinogen was dissolved in 1% L-Arginine, 1% L-Lysine and 0,94 PEU/ml aprotinin to obtain a 60 mg/ml solution (pH between 6.5 and 7.5). Lyophilized thrombin was reconstituted with a 275 mM CaCl_2_ in H_2_Od at a 1250 U/ml concentration (pH between 6.5 and 8.0).

The s-IPN PEtU-PDMS/fibrin combined scaffold was manufactured in the form of a flat membrane by a previously described spray, phase-inversion technique [22] in a Class 100 clean room. Briefly, the manufacturing process consisted of two phases: in the first phase, a 2.5% s-IPN PEtU-PDMS solution and H_2_Od were employed; in the second phase, a 1% s-IPN PEtU-PDMS solution and a 25 U/ml thrombin solution were sprayed simultaneously. The thrombin-entrapped s-IPN PEtU-PDMS scaffold was punched to obtain round samples of 1 cm^2^ area and placed into the bottom of a 24-well culture plate. Finally, a fibrinogen solution (20 mg/ml) was added to each well (600 µl/well). Complete polymerization reaction into fibrin was achieved after an overnight incubation at 37°C. The round samples were stored in physiological saline solution at 4°C and used for the experiments within 1 week. Sterile conditions were maintained throughout all steps of the fibrin-based scaffold fabrication.

The sterility of the s-IPN PEtU-PDMS/fibrin combined scaffold was assessed incubating scaffold samples in Mueller–Hinton broth (Oxoid S.p.A., Milano, Italy) under agitation at 37°C for 5 days. No clouding of the broth was observed indicating the absence of bacterial contamination.

### Light microscopy

The s-IPN PEtU-PDMS/fibrin combined scaffold was stained with Ponceau red dye and observed by a stereo-microscope (SZH10 microscope, Olympus Optical Co., Tokyo, Japan) to verify the presence of a homogenous fibrin coating. In brief, samples were dipped in a staining solution (0.5% w/v in 1% acetic acid) for 5 min at room temperature and then rinsed in distilled water to remove the excess dye. Moreover, cross-sectional images acquired at 60× original magnification were analyzed using Axiovision Rel 4.6 image analysis software (Carl Zeiss, Jena, Germany) to measure fibrin and PEtU-PDMS layers thickness of the fibrin-based scaffold; six random measurements were performed for each image.

### Scanning electron microscopy

The structure of the fibrin side of the s-IPN PEtU-PDMS/fibrin combined scaffold was observed using a scanning electron microscope (SEM, Jeol 5600, Jeol Italia, Milan, Italy) after gold-palladium metallization by a sputter coater system (Sputter coater S150B, Edwards, Irvine, CA, USA). SEM microphotographs were taken at 1000× and 20.000× magnifications with a 18–20 kV acceleration voltage. The images acquired at 20.000× were analyzed by the computerized image analysis system (Axiovision Rel 4.6, Carl Zeiss, Jena, Germany) to quantitatively determine fibrin fibers mean diameter; six random measurements were performed for each image.

### Stem cell culture

According to the guidelines of the Ethical Committee of the FBF S. Peter Hospital, hAMSCs were obtained from human term placenta from healthy women with written informed consent and generally processed immediately. Isolations are usually performed with term amnion dissected from the deflected part of the fetal membranes to minimize the presence of maternal cells. Homogenous hAMSC populations can be obtained by a two-step procedure: pieces of amniotic membrane were minced and treated for 15 min with 0.25% trypsin-EDTA solution to remove human amniotic epithelial cells (hAEC), then the supernatant was discarded and the remaining mesenchymal cells underwent a second digestion with 0.25% trypsin-EDTA, 10 U/ml DNAse I (Sigma-Aldrich, St. Louis, USA) and 0.1% collagenase IV (Sigma-Aldrich) solution in Dulbecco's modified Eagle's medium (DMEM) [72]. The supernatant was transferred to a fresh tube, neutralized with Fetal Bovine Serum (FBS) then spun at 1500 rpm for 10 min. Each pellet was re-suspended in 5 ml of culture medium containing: DMEM, 10% FBS, penicillin (100 U/ml) and streptomycin (100 µg/ml) (PBI international Milano, Italy), EGF (10 ng/ml, ImmunoTools, Friesoythe, Germany) and β-mercaptoetanolo (55 µM, Sigma-Aldrich). The yield from term amnion is about 1 million of hAMSCs per gram of tissue [72]. hAMSCs were cultured on plastic Petri dishes at 37°C in a humidified atmosphere containing 5% CO_2_. Non-adherent cells were removed after 1 week and when the culture reached 90% confluence, cells were recovered using 0.25% Trypsin-EDTA and plated in Petri dishes at a density of 1×10^4^ cells/cm^2^; the medium was subsequently changed every 3 days.

Cell characterization by flow cytometry revealed the presence of mesenchymal markers (CD73, CD105, CD29, CD44, CD54, ImmunoTools) and the absence of hematopoietic markers (CD34, CD31, CD45, ImmunoTools) on hAMSC membrane. The immunophenotypical profile of hAMSC corresponded to the one reported in literature for bone marrow MSCs [73], [74].

At passage 3, 5×10^4^ stem cells were seeded on the s-IPN PEtU-PDMS/fibrin combined scaffold and in plastic Petri dishes for cell metabolic activity and proliferation assays. hAMSCs were exposed to nitric oxide (NO) or non-exposed to NO (control) and maintained in culture for up to 14 days in a humidified incubator (37°C, 5% CO_2_). The cells were exposed to NO by exogenous supplementation of *S*-nitroso-*N*-acetylpenicillamine (SNAP, 0.4 µM, Invitrogen) at the starting time of the experiment, replaced on day 2 and maintained until day 4 [59]. Then, cell cultures were maintained in medium without NO agent, and replaced with fresh medium every 2 days, until 14 days. These cells can be kept until passages 5–10.

### WST-1 assay

Quantification of hAMSCs metabolic activity was performed by a colorimetric assay based on the reduction of water-soluble tetrazolium salts (Cell Proliferation Reagent WST-1; Roche Diagnostics, Basel, Switzerland).

Exponentially growing hAMSCs were seeded on the s-IPN PEtU-PDMS/fibrin combined scaffold and in plastic Petri dishes at a density of 5×10^4^ cells/scaffold and 5×10^4^ cells/Petri dish and cultured for up to 14 days in a humidified incubator (37°C, 5% CO_2_). Cells were differentiated by exogenous supplementation of *S*-nitroso-*N*-acetylpenicillamine (SNAP, 0.4 µM). WST-1 reagent diluted to 1∶10 was added in the medium of hAMSCs at 1, 2, 3, 5, 6, 10 and 14 days following plating, and after an incubation of 2 h in a humidified atmosphere, the supernatants (100 µl) of hAMSCs were put in 96-well plates and analyzed by means of the formazan dye. Quantification of the formazan dye produced was performed by absorbance measurement at 450 nm with a scanning multiwell spectrophotometer (Biotrack II; Amersham Biosciences, Little Chalfont, UK).

### Bromodeoxyuridine incorporation assay

Exponentially growing hAMSCs were seeded on the s-IPN PEtU-PDMS/fibrin combined scaffold and in plastic Petri dish at a density of 5×10^4^cells/scaffold and 5×10^4^ cells/Petri and cultured for up to 14 days. Stem cells were treated or not treated with SNAP to induce cardiac differentiation. 10 mM BrdU was added in the medium of hAMSCs at 1, 2, 3, 5, 6, 10 and 14 days after plating and maintained for 18 h in culture. Cells were then fixed and incubated for 30 min at 37°C with the anti-BrdU antibody (1∶100; Cell Proliferation Kit; Roche Diagnostics). After incubation with 2,20-Azino-bis(3-ethylbenzothiazoline-6-sulfonic acid) for 30 min, the absorbance of 100 µl of supernatant was measured in an ELISA reader at 450 nm.

### Real-time quantitative reverse transcriptase polymerase chain reaction analysis

Total RNA was extracted at 4,7, 9 and 14 days from control and SNAP treated hAMSC grown up on the s-IPN PEtU-PDMS/fibrin combined scaffold, using TRIzol Reagent (Invitrogen). An average 1–5 µg total RNA per scaffold of cultured cells was obtained. One microgram of total RNA was used to synthesize firststrand cDNA with random primers, using 100 U of ImProm-IITM RT–PCR kit (Promega, Madison, WI, USA). The quantification of all gene transcripts was carried out by real-time quantitative reverse transcriptase polymerase chain reaction (RT–PCR). Experiments were conducted to contrast relative levels of each transcript and endogenous control GAPDH in every sample. Gene expression was presented using the (2^−DCt^) method, described by Livak and Schmittgen [75], where DCt = (average target Ct- average GAPDH Ct). We performed a validation experiment to prove that the amplification efficiency on target genes and reference GAPDH was equal. RT-PCR was performed with Sybr Green I Mastermix, using an ABI PRISM 7000 Sequence Detection System (Applied Biosystems, Foster City, CA, USA). Each reaction was run in triplicate and contained 0.5–1 µL of cDNA template along with 250 nM primers in a final reaction volume of 25 µL. The genes investigated were NK2 transcription factor related, locus 5 (nkx2.5) and cardiac troponin I (TnI) for cardiac differentiation, VEGF and KDR for endothelial differentiation. The annealing temperatures for all primers used in this study are 60°C and the sequences are:

kx2.5: 5′-CAGCGACCCCGACCCAG-3′; 5′-GCTTCCTCCGCCGTCGC-3′,

TnI: 5′-GGACAAGGTGGATGAAGAGA-3′; 5′-AGGGTGGGCCGCTTAAACT-3′,

VEGF: 5′-CTTGGGTGCATTGGAGCCT-3′; 5′-CTGCGCTGATAGACATCCAT-3′,

KDR: 5′-CAGCATCACCAGTAGCCAG-3′; 5′-TTATACAGATCTTCAGGAGCTT-3′,

GAPDH: 5′-CATCATCTCTGCCCCCTCT-3′; 5′-CAAAGTTGTCATGGATGACCT-3′

Cycling parameters were: 50°C for 2 min, 95°C for 10 min (to activate DNA polymerase), then 40–45 cycles at 95°C for 15 s and 60°C for 1 min. Melting curves were performed using Dissociation Curves software (Applied Biosystems) to ensure that only a single product had been amplified. As negative controls, reactions were prepared in which RNA or reverse transcriptase had previously been omitted during reverse transcription.

### Immunofluorescence analysis

hAMSCs were seeded on the s-IPN PEtU-PDMS/fibrin combined scaffold at a density of 5×10^4^ cells per scaffold and cultured for 14 days, in absence (CTR) or in presence (NO) of differentiating agent, as shown in the above protocol. At 1, 4, 7 and 14 days, hAMSCs were fixed in paraformaldehyde 4% at 4°C for 10 min, washed twice in Ca^2+^/Mg^2+^-free PBS and permeabilized at room temperature for 15 min (0.1% Triton X-100, 1% BSA; Sigma-Aldrich). hAMSCs were incubated with phalloidin TRIC (tetrametyl rhodamine isothiocyanate) conjugated (1∶100), an anti-actin toxin (Sigma-Aldrich), in a blocking buffer for 1 hour or with primary antibody Vimentin (1∶200) (abcam, Cambridge, UK). After washing in PBS containing 0.1% Triton X-100 and 1% BSA, the cells were incubated with the anti-mouse secondary antibody (1∶100) (Chemicon, Billerica, MA, USA). Cells were washed three times with PBS, stained for nuclei localization with Hoechst 33342 (trihydrochloride-trihydrate) and examined. The scaffolds were overturned on cover glasses and tested by indirect immunofluorescence for the presence of vimentin and actin filaments. Fluorescence measurements were obtained using an inverted microscope (Olympus IX51, RT Slider SPOT - Diagnostic instruments, Sterling Heights, MI, USA) equipped with a 20× objective and with a cooled CCD camera (Spot RT Slider, acquisition rate five frames per second, full frame; Diagnostic Instruments, Sterling Heights, MI, USA). No significant fluorescent signal was detectable with the secondary antibody alone. Densitometric analysis of the vimentin immunofluorescence was performed with Imagej Software.

### Statistical analysis

Statistical analysis of the data was performed using Student's t-test, with P≤0.05 as the minimum level of significance. For WST-1 and Bromodeoxyuridine incorporation assays the results were obtained by six independent experiments (n = 6), while for the values obtained by RT-PCR analysis each bar represents the ±SD of three independent experiments (n = 3). Data are shown as mean ± standard deviation (SD).
